# A Solitary Wave-Based Sensor to Monitor the Setting of Fresh Concrete

**DOI:** 10.3390/s140712568

**Published:** 2014-07-14

**Authors:** Piervincenzo Rizzo, Xianglei Ni, Somayeh Nassiri, Julie Vandenbossche

**Affiliations:** 1 Laboratory for Nondestructive Evaluation and Structural Health Monitoring Studies, Department of Civil and Environmental Engineering, University of Pittsburgh, 3700 O'Hara Street, 729 Benedum Hall, Pittsburgh, PA 15261, USA; 2 INTECSEA, WorleyParsons Group, Floating System Department, 575 N. Dairy Ashford, Houston, TX 77079, USA, E-Mail: xiangleini@gmail.com; 3 Department of Civil & Environmental Engineering, Washington State University, 405 Spokane Street, Sloan Hall 35, Pullman, WA 99164, USA, E-Mail: snassiri@wsu.edu; 4 Department of Civil and Environmental Engineering, University of Pittsburgh, 3700 O'Hara Street, 719 Benedum Hall, Pittsburgh, PA 15261, USA; E-Mail: jmv7@pitt.edu

**Keywords:** highly nonlinear solitary waves, nondestructive evaluation, concrete hydration, initial and final set

## Abstract

We present a proof-of-principle study about the use of a sensor for the nondestructive monitoring of strength development in hydrating concrete. The nondestructive evaluation technique is based on the propagation of highly nonlinear solitary waves (HNSWs), which are non-dispersive mechanical waves that can form and travel in highly nonlinear systems, such as one-dimensional particle chains. A built-in transducer is adopted to excite and detect the HNSWs. The waves are partially reflected at the transducer/concrete interface and partially transmitted into the concrete. The time-of-flight and the amplitude of the waves reflected at the interface are measured and analyzed with respect to the hydration time, and correlated to the initial and final set times established by the penetration test (ASTM C 403). The results show that certain features of the HNSWs change as the concrete curing progresses indicating that it has the potential of being an efficient, cost-effective tool for monitoring strengths/stiffness development.

## 1. Introduction

The chemical reaction between water and the cement is referred to as hydration. The mixture progressively develops as the hydration process continues and eventually initial set is achieved. The initial set is the time between the first contact of water with the cement grains and the time at which workability is lost. Final set is the time required for the fresh concrete transform from plastic into a rigid state. The initial and final set times are established in accordance with ASTM C403 [1] and correlate to the time when the mortar can resist the penetration of multi-sectional metal needles with applied pressure levels of 3.4 and 24.6 MPa, respectively. These pressures characterize the resistance of a mortar sample, obtained through wet sieving of the concrete mixture, against the penetration.

At final set, measurable mechanical properties start to develop in concrete and continue to grow progressively. Knowing the rate of strength development at early ages is critical in establishing the timeframe for construction-related activities, such as when to saw joints or to open the roadway to traffic for a newly-placed concrete pavement. Concrete samples are cast so that strength testing can be performed to determine when sufficient strength has been achieved to open the roadway to traffic [2]. The use of a nondestructive test method for estimating the strength would eliminate the need for making and testing the concrete samples and would allow for frequent monitoring without concern for the amount of strength samples available. Special care is also needed when defining the window for sawing the joints for a jointed plain concrete pavements (JPCPs). While late sawing of the joints late can result in uncontrolled cracking of the concrete requiring expensive repairs, sawing too early can result in excessive spalling making it difficult to keep the joint well sealed.

Since the hydration is an exothermic reaction, the concrete mixture has a specific heat signature. One approach commonly employed for monitoring strength gain in the concrete is to monitor the temperature of the concrete as a function of time. The maturity of the concrete can then be estimated by integrating the area between the temperature-time curve and a datum. Maturity-strength relationships can then be established for a specific mixture so that the strength of the mix can be estimated by monitoring the heat of the mixture as is hydrated. Unfortunately, the maturity-strength relationship is very specific to the concrete mixture proportions for which it was developed. It is no longer representative if the concrete mixture proportions or the source of the constituents within the mixture are varied. The disadvantage of this method is that preliminary testing must be performed to establish this maturity-strength relationship. Also, the relationship must be re-established if at any time the source or quantity of a particular constituent in the concrete mixture is changed. For these reasons, direct but simple nondestructive measurements are needed to monitor the development of the mechanical properties in newly-placed concrete pavements. However, nondestructive measurements can be also utilized to: determine the ultimate strength of concrete or its elastic modulus; establish the time when forms can be removed; decide when prestress can be added to bridges; advance a segmental bridge construction.

**Figure 1.**
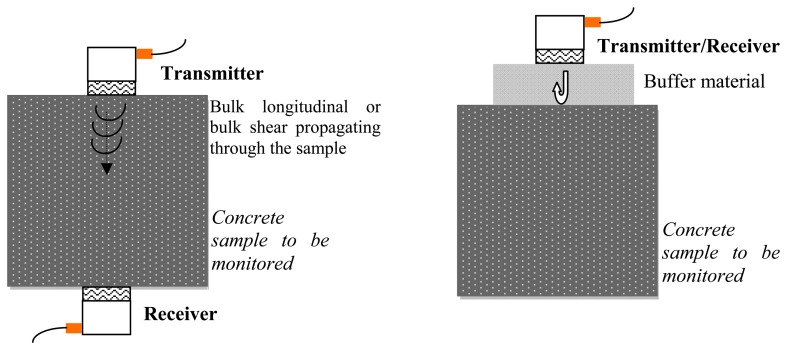
(**a**) Through-transmission; (**b**) Pulse-echo configuration.

Nondestructive evaluation (NDE) techniques have been widely adopted over the past two decades for monitoring the development of strength in fresh concrete. Most of these techniques are based on the measurement of the velocity of linear bulk ultrasonic waves propagating through a concrete sample. Traditionally, commercial transducers are used to generate longitudinal [3–11], or both longitudinal and shear waves [12]. Parameters such as wave speed and attenuation are measured and empirically correlated to the material properties. This approach is usually referred to as the ultrasonic pulse velocity (UPV) method. To obtain an acceptable signal-to-noise ratio, longitudinal wave transducers cannot be used to generate transverse waves and *vice versa*. Thus, in order to use both shear and longitudinal waves; at least four transducers are required. If the access to the back wall of the sample is impractical, the wave reflection method can be adopted. In this approach, the amplitude of the shear waves [12–19] or the longitudinal waves [20,21], or both [22] at an interface between a buffer material, typically a steel plate, and the concrete is monitored over time. The amount of wave reflection depends on the reflection coefficient, which in turn is a function of the acoustical properties of the materials that form the interface [12]. The use of bulk waves is schematized in Figure 1. Figure 1a shows the through-transmission scheme where two longitudinal or shear transducers are used to transmit and receive bulk waves. Drawbacks of this scheme are: (1) four transducers are necessary to exploit both modes; (2) the access to the back-wall is needed and this is not always possible; (3) the exact distance between the transducers pair must be known to accurately measure the wave speed exactly; (4) the contact conditions between the transducers and the concrete surface must be kept constant to avoid any mislead assessment of the amplitude. Figure 1b schematizes the pulse-echo configuration where either one longitudinal or one shear transducer is used in dual-mode; *i.e.*, as both transmitter and receiver. As the wave speed in the buffer material is constant irrespective of the concrete age; only one wave parameter (the amplitude) can be exploited unless two transducers (one S- and one L-) are used.

Besides the use of bulk waves, other NDE technologies proposed to observe the growth of mechanical properties in concrete are based on guided ultrasonic waves [23], surface wave [24,25], fiber optics [26] and acoustic emission [27]. Boulay *et al.* [28] compared some static and ultrasonic methods aimed at measuring changes in the stiffness of concrete at early age. The static approaches were based on classical loadings in hardened concrete and in cycling loadings using two different machines; the nondestructive methods were based on the natural resonant frequency of a composite beam, ultrasonic measurements using classical equipment, and ultrasonic measurements using sensors embedded in the test samples.

In this paper we present the proof-of-principle of a novel NDE paradigm to monitor the strength development of hydrating concrete. The method is based on the use of a transducer described for the first time in [29] able to generate and detect highly nonlinear solitary waves (HNSWs) that are nondispersive mechanical waves that can form and travel in highly nonlinear systems, such as a closely packed chain of elastically interacting particles, also called granular crystals [30–32]. The most common way to induce solitary waves is by impacting the first particle of the chain with a striker. The impact velocity and the mass of the striker determine the characteristics of the traveling HNSWs in terms of number of forming pulses, pulses speed, amplitude, and duration. In this study an actuator/sensing system, hereafter indicated as the HNSW transducer, was used to generate solitary waves and detect their reflection at the transducer/concrete interface.

The interaction of the HNSWs with linear systems was studied earlier [33–38]. Manciu and Sen [33] investigated the wave reflections from rigid wall boundaries. Falcon *et al.* [34] studied the fragmentation of a chain of particles when impacting a fixed wall. Job *et al.* [35] evaluated the collision of a single solitary wave with elastic walls with various hardnesses. Yang *et al.* [36] studied in detail the interaction of HNSWs with uniform and composite elastic media. It was shown that the formation and propagation of reflected HNSWs are highly dependent on the elastic modulus and geometry of the adjacent medium. Ni *et al.* [37] analyzed experimentally the interaction of HNSWs with a cement paste employing the same transducer used in the present study. The results were then compared with those obtained using a numerical model developed to simulate the interaction between the HNSWs and the underlying system. Finally, Cai *et al.* [38] reported on the interaction of HNSWs with slender beams.

With respect to previous works on stress waves for the NDE of concrete, the differences of the proposed approach are: (1) it exploits the propagation of HNSWs in granular systems; (2) it employs a cost-effective actuator/sensor in a combined form; (3) it measures several waves' parameters (time of flight, speed and amplitude of one or two waves) that can be eventually used to correlate few concrete variables; (4) it does not require, unlike UPV method, the exact knowledge of the distance between a transmitter and a receiver and does not require the access to the sample back-wall. The proposed HNSW-based method may resemble the Schmidt hammer, which can be used to estimate the hardness and strength of concrete [39] and rock [40]. The Schmidt hammer is a spring-driven steel hammer that hits the specimen with a defined energy. Part of the impact energy is absorbed by the plastic deformation of the specimen and transmitted to the specimen, and the remaining impact energy is rebounded. The rebound distance is dependent on the hardness of the specimen and the conditions of the surface. The harder is the surface, the shorter is the penetration time or depth, hence the higher is the rebound. Based on the knowledge available in the open literature, there are several differences between the Schmidt hammer and the proposed HNSW-based method that can be summarized as follows: the Schmidt hammer can be only used to test hardened material, but the HNSW approach can be applied also onto fresh concrete; only one parameter, the rebound value, is used in the Schmidt hammer test, while multiple HNSWs features can, in principle, be exploited to assess the condition of the underlying material; the reliability and repeatability of the Schmidt hammer are not guaranteed especially when the elastic modulus of the sample is low [40] while recent studies [32,37,41] have shown that HNSWs pulses can be generated with high confidence of repeatability; the Schmidt hammer may induce plastic deformation or microcracks to the specimen, while the HNSW approach is purely nondestructive as there is not mechanical impact on the material under testing. Finally, it is worth noting the following differences between the present paper and ref. [37]. In this study: (1) we monitored a concrete instead of plaster cement; the two materials are quite different in terms of composition, curing time, and engineering applications; (2) we tested a standard concrete cylinder instead of a cup filled with plaster which is almost one order of magnitude smaller; (3) we utilized a transducer automatically governed by means of a National Instruments PXI running under LabView. A user friendly interface was created such that the whole experiment could be conducted without the presence of the operator. In [37] instead, the transducer was manually driven and the time signals were digitized by means of an oscilloscope; each measurement required the action of the operator; (4) we compared the results of the HNSW-based method to the results of the standard ASTM C403.

The paper is organized as follows. Next section describes the underlying basis of HNSWs following the analytical formulation adopted in [37,42]. The overall methodology for the NDE of concrete is also described. Then, the experimental results are presented. Finally the conclusions section summarizes the findings and it highlights the future studies that should be carried out to fully prove the effectiveness of the proposed NDE methodology.

## 2. Background

**Figure 2.**
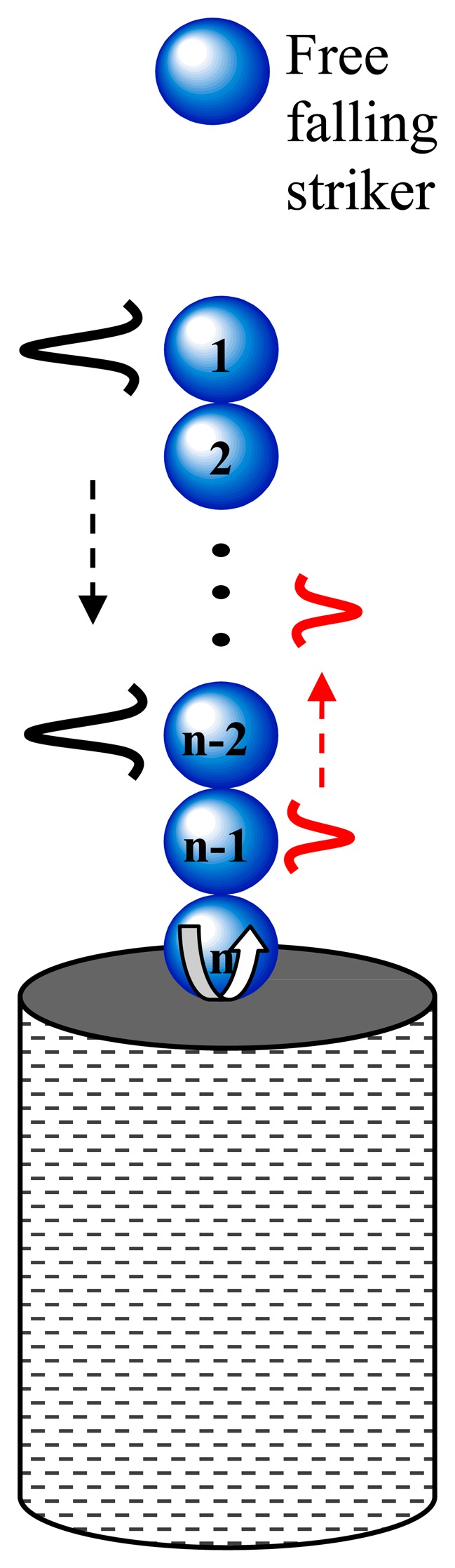
General scheme of structural assessment by means of HNSWs.

Figure 2 illustrates the general principles of the proposed NDE technique. A HNSW-based transducer, here schematized with a chain of spherical particles, is in contact with fresh concrete. A thin (<1 mm thick) aluminum sheet may be placed in between the transducer and the concrete to prevent the penetration of the bottom sphere into the fresh concrete. The impact of a striker, made of a particle of equal size and mass of the other particles composing the chain, generates a single pulse that propagates through the chain.

In general, in a chain of spherical particle the interaction between two adjacent beads is governed by the Hertz's law [43,44]:

(1) $$F=A\delta^{3/2}$$

which establishes a relationship between the compression force *F* of granules and the closest approach *δ* of particle centers. In Equation (1) the coefficient *A* is given by:

(2) $$A=\frac{E\sqrt{a}}{3\left(1-\nu^{2}\right)}$$

where *a* is the diameter of the beads, and *ν* and *E* are the Poisson's ratio and Young's modulus of the material constituting the particles, respectively.

The combination of the nonlinear interaction (Equation (1)) and a zero tensile strength in the chain of spheres leads to the formation and propagation of compact solitary waves [44]. When the wavelength is much larger than the particles' diameter, the speed of the solitary waves *V_S_* depends on the maximum dynamic strain *ξ_m_* [44] which, in turn, is related to the maximum force *F_m_* between the particles in the discrete chain [32]. When the chain of beads is under a static pre-compression force *F_0_*, the initial strain of the system is referred to as *ξ_0_*. It should be noted that in configurations like the one shown in Figure 2, the pre-compression is given by the self-weight of the chain. The speed of the solitary wave *V_s_* has a nonlinear dependence on the normalized maximum strain *ξ_r_* = *ξ_m_*/*ξ_0_*, or on the normalized force *f_r_* = *F_m_*/*F_0_*, expressed by the following equation [32]:

(3) $$V_{S}=c_{0}\frac{1}{\left(\xi_{r}-1\right)}\times {\left\{\frac{4}{15}[3+2\xi_{r}^{5/2}-5\xi_{r}]\right\}}^{1/2}=0.9314{\left(\frac{4E^{2}F_{0}}{a^{2}\rho^{3}{\left(1-\nu^{2}\right)}^{2}}\right)}^{1/6}\frac{1}{\left(f_{r}^{2/3}-1\right)}{\left\{\frac{4}{15}[3+2f_{r}^{5/3}-5f_{r}^{2/3}]\right\}}^{1/2}$$

where *c_0_* is the wave speed in the chain initially compressed with a force *F_0_* in the limit *f*_r_ =1, and *ρ* is the density of the material. When *f_r_* (or *ξ_r_* ) is very large, Equation (3) becomes:

(4) $$V_{S}=0.6802{\left(\frac{2E}{a\rho^{3/2}(1-\nu^{2})}\right)}^{1/3}F_{m}^{1/6}$$

which represents the speed of a solitary wave in a “sonic vacuum”. The shape of a solitary wave with a speed *V_s_* in a “sonic vacuum” can be closely approximated by [44]:

(5) $$\xi =\left(\frac{5V_{s}^{2}}{4c^{2}}\right)\cos^{4}\left(\frac{\sqrt{10}}{5a}x\right)$$

where:

(6) $$c=\sqrt{\frac{2E}{\pi \rho (1-\nu^{2})}}$$

and *x* is the coordinate along the wave propagation direction.

In this study, the underlying research hypothesis is that the changes of the mechanical properties of concrete during hydration, alter the contact stiffness of the chain/material interface. This alteration is sensed by the HNSW transducer by monitoring certain features of the solitary waves. In the proposed NDE approach we monitor the waves reflected from the transducer/cement interface using instrumented particles, herein indicated as sensor beads, inserted in the chain. The characteristics of the reflected pulses in terms of their amplitude, time-of-flight (TOF), and speed are correlated to the progression of the hydration process. When a single pulse reaches the interface with the material to be tested, the pulse is partially reflected. When the pulse interacts with a “soft” medium, secondary reflected solitary waves (SSW) form in the granular crystal, in addition to the primary reflected solitary waves (PSW) [34,36,37,40]. In this study, we hypothesize that these reflected waves are strongly influenced by the concrete mechanical properties, and in particular, they can identify the initial and final set of the mixture. We characterize the wave reflection properties measuring the TOF, the amplitude of the primary reflected solitary wave (ARP), and the amplitude of the secondary reflected solitary wave (ARS). Here, the TOF denotes the transit time at a given sensor bead in the granular crystal between the incident and the reflected waves. We define the ARP as the ratios of the PSW amplitude divided by the incident solitary wave amplitude and the ARS as the ratio between the SSW amplitude and the incident wave amplitude.

**Figure 3.**
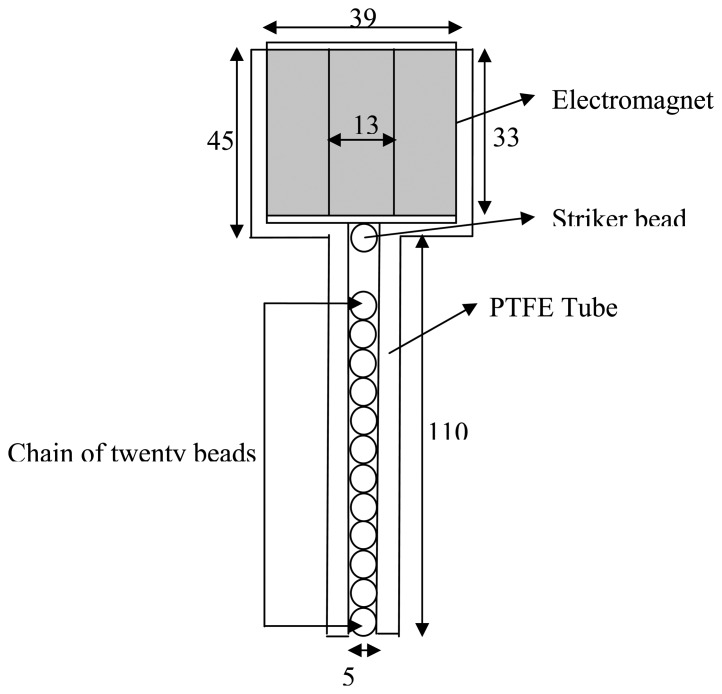
Schematic of the HNSW-transducer used in the study (dimensions are expressed in mm).

In order to generate and detect the HNSWs a cost-effective transducer recently developed [29] was used. The transducer is schematized in Figure 3 and it consisted of a polytetrafluoroethylene (PTFE) tube with an inner diameter of 4.8 mm, filled with −20 type-302 stainless steel beads. The diameter and the mass of each sphere were 4.76 mm and 0.45 g, respectively. Two piezo-gages made from lead zirconate titanate were embedded inside two of the steel particles. Each piezo-gauge was equipped with nickel-plated electrodes and custom micro-miniature wiring. The sensor beads were positioned along the chain at the 11th and 16th position from the top. The striker was a low-carbon steel bead with a diameter of 4.76 mm and mass of 0.45 g. The transducer's ability to generate repeatable solitary pulses was originally presented in [28] and proven in later studies [37,41].

The low-carbon steel was chosen in order to ease the movement of the striker by means of an electromagnet which, in turn, was controlled by a National Instrument–PXI unit running in LabView.

The position of the sensor beads in the chain takes into account a few factors. First, the consolidation of the HNSWs is complete approximately few beads away from the location of the impact. Second, the sensor bead cannot be located too close to the end of the chain otherwise we would measure the interference of the reflected and incident solitary waves. Third, the distance between two sensor beads should be as large as possible to minimize the relative error associated with the measurement of the spatial distance between the sensors. However, this distance must be kept reasonable to avoid using an unpractical long transducer.

## 3. Experimental Test

To prove the feasibility of the proposed NDE method, an experiment was performed in the laboratory on a 15.24 cm by 30.5 cm cylindrical concrete specimen. The mixture for the concrete is summarized in Table 1. The water/cement ratio was equal to 0.42 and the 28 day compressive strength was 27.1 MPa. This value was determined by averaging the results from three cylinders. The concrete cylinder was cast at 8:30 a.m. in the laboratory.

**Figure 4.**
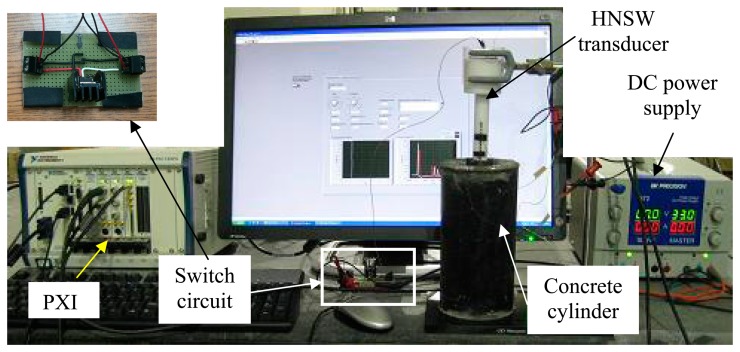
Photo of the experimental setup. Top left: close-up view of the switch circuit.

The concrete was mixed at a batch plant approximately seven miles away from the laboratory. The time water contacted the cement was estimated to be around 8:00 a.m. Therefore, one half-hour was added to the duration of the test to account for the travel time from the batch plant to the laboratory. A 40 × 40 × 0.254 mm aluminum sheet and the actuator were placed on top of the specimen five minutes after casting the specimen. A photo of the setup is shown in Figure 4.

The experiment began at 10:05 AM, immediately after placing the transducer above the sample. Ten measurements were taken every 15 min for a duration of ten hours. The initial and final set times were established by performing the ASTM C 403 on mortar wet sieved from the concrete sample.

**Figure 5.**
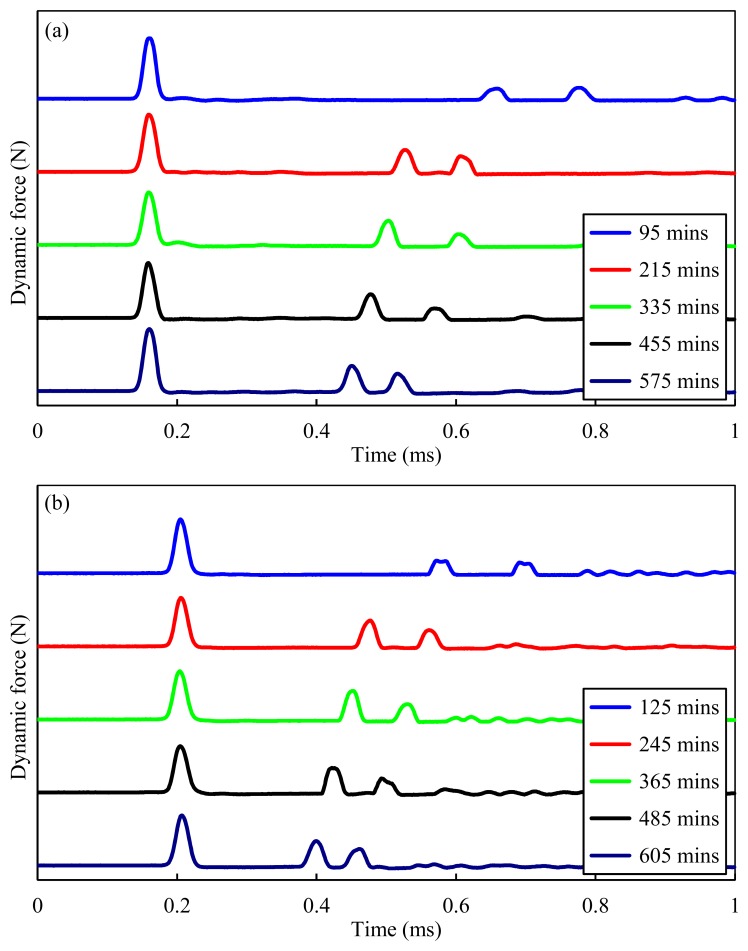
Force profile of the HNSWs waveforms recorded at different ages of concrete. Measurements taken at the (**a**) 11th bead and (**b**) 16th bead.

Figure 5 shows the temporal force profiles computed at both sensor particles, when an incident solitary wave interacted with concrete at five different instances. The profiles associated with the two sensor beads and displayed in Figure 5a,b, respectively, are purposely presented with a time offset of 30 min with respect to each other, to demonstrate that both sensors were equally efficient across the whole experiment. The signals obtained for each run are shifted vertically for better comparisons. Three pulses are visible for each instance. The first pulse represents the incoming solitary wave arriving at the sensor bead, while the second and third pulses are the PSW and SSW, respectively. It is noticeable that the TOFs of both the SSWs and PSWs are strongly dependent on the sample's age. As the hydration progresses, the sample's stiffness increases and the TOF of the SSWs and PSWs decreases. Moreover, the amplitude of the PSW increases and the TOF of the PSW decreases.

**Figure 6.**
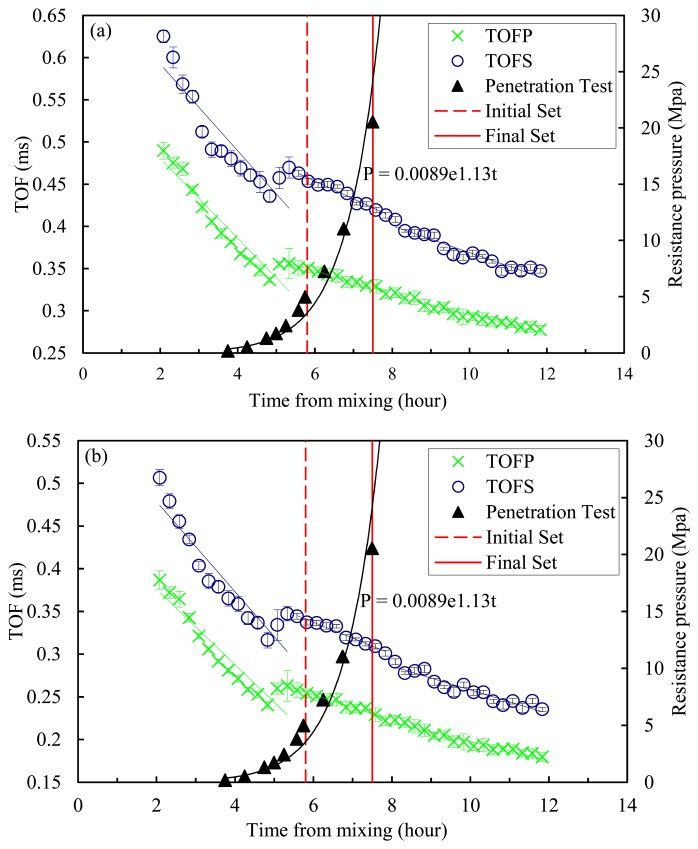
TOF of the PSW (TOFP) and SSW (TOFS) measured from the (**a**) 11th and (**b**) 16th bead in the HNSW transducer and penetration resistance as a function of time.

**Figure 7.**
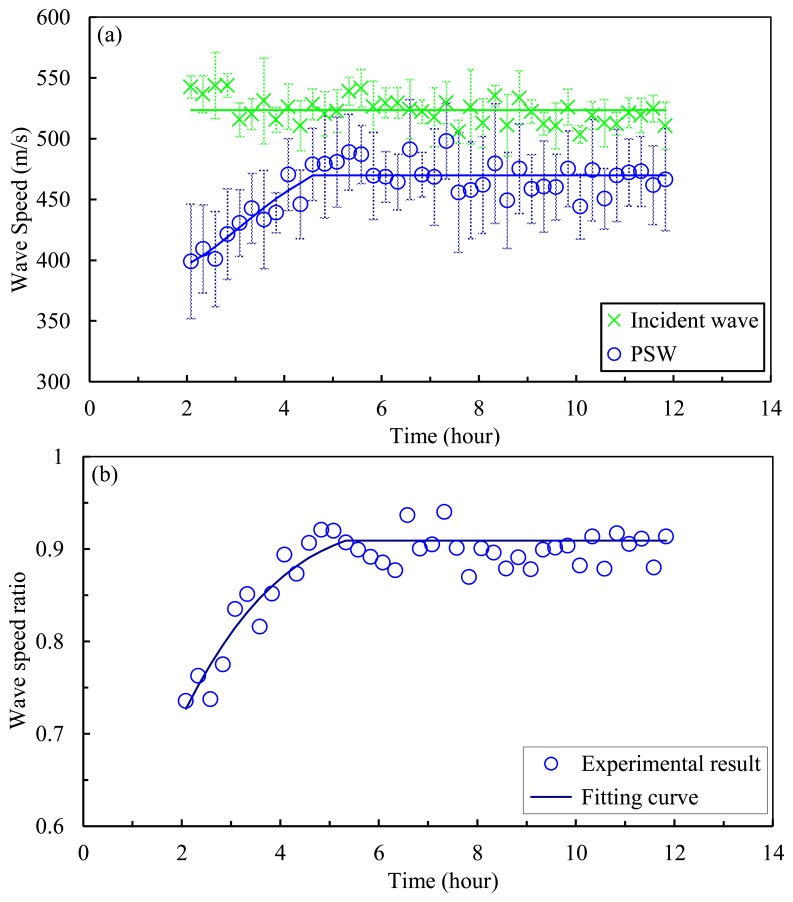
(**a**) Experimental results of wave speed of incident HNSW and PSW; (**b**) The ratio of the wave speed of PSWto that of incident HNSW.

**Figure 8.**
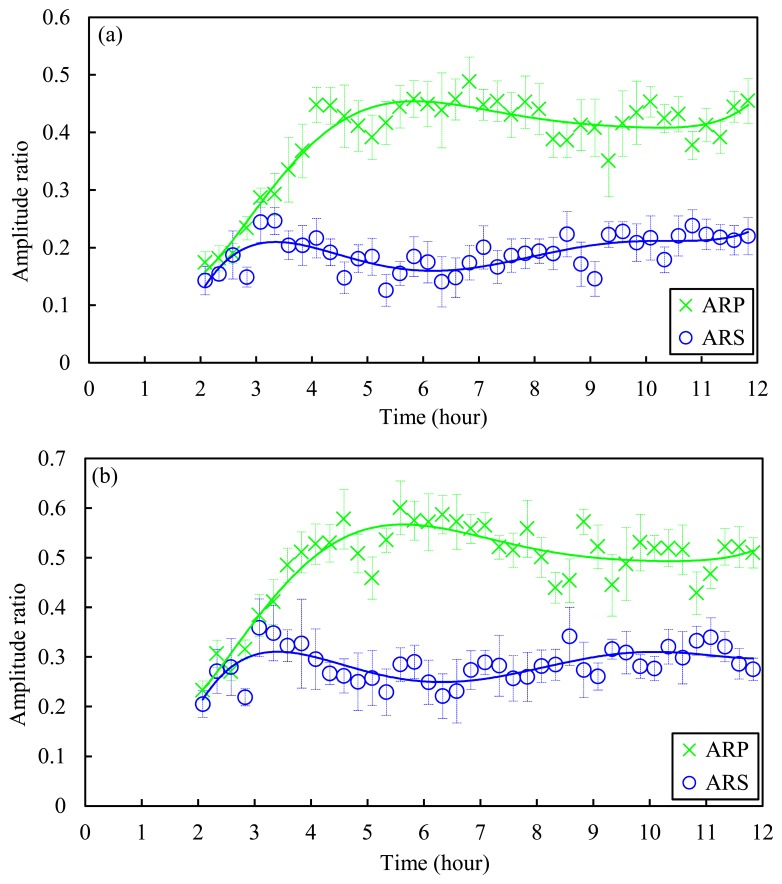
Experimental results of amplitude ratio of PSW (ARP) and SSW (ARS) as a function of time measured from the (**a**) 11th and (**b**) 16th bead in the HNSW transducer.

In order to quantify the effect of aging on some characteristics of the solitary waves, Figures 6, 7 and 8 are reported. Figure 6 shows the measured TOFs of both the SSW (green crosses) and the PSW (blue circles) as a function of the hydration time. Each data point in the figure indicates the mean value of the ten experimental measurements, and the vertical error bars represent the 95.5% confidence interval. Figures 6a refers to the measurement associated with the 11th particle whereas Figure 6b refers to the bottom sensor site, *i.e.*, the sensor bead closer to the chain/concrete interface. In both figures, the small value of the standard deviation demonstrates the capability of the transducer to generate repeatable pulses. The penetration resistance as established by the ASTM C 403 is superimposed. The slope of the TOF curves indicates the presence of a two-stage behavior. In the first stage, lasting about 300 min, the TOF values of both the PSW and SSW show a rapid drop. Although the transition between the two stages is close to the time of initial set established with the penetration test, there is not a perfect agreement between the novel and the conventional methodology. It is believed that the sieving process used to extract the mortar sample from the concrete for the penetration test, has different local characteristics with respect to the concrete sample monitored with the solitary waves and therefore some degree of discrepancy should be expected.

It should be also mentioned that the penetration test measures the penetration resistance and defines the initial and final set using two arbitrarily-chosen values, but the HNSW-based method measures the “effective” stiffness of the concrete sample at least in the local region underneath the transducer.

After 300 min, the decrease in the TOF continues with a considerably different rate (more gradual) until the end of the experiment. Around 485 min (8 h 5 min) there is a slight decrease in the gradient which could be associated with the time of final set, 460 min (7 h 40 min), determined by the penetration test. However this second slope change is not as visible as the first one and additional experiments are necessary to demonstrate if the TOF can be used to identify the final set.

Figure 7a shows the wave speed of the incident solitary wave (green crosses) and of the PSW (blue circles) as a function of the concrete age. The speed is calculated by dividing the distance of the sensor beads by the measured time of arrival at these sensor beads. Similar to the TOF, the speed of the PSW increases 17% over the first 300 min and remain constant thereafter. As expected, the speed of the incident wave velocity remains constant throughout the experiment. The small scatter in the speed of the incident wave can be attributed to the energy of the striker at the moment of the impact with the chain [32], determined by the friction with the inner tube. Variation in energy results in different momentum transferred to the chain which, in turn, affects the amplitude and speed of the generated solitary waves [29–32].

In order to minimize any effect associated with the variation of the incident wave speed, the speed of the primary reflected wave was normalized with respect to the speed of the incident wave. The results are presented in Figure 7b. The normalized speed increases by approximately 30% over the first 300 min and then remains constant as the hydration progresses.

By comparing Figure 7 with Figure 6, it was observed that the wave velocity becomes saturated after 5 h, while the TOF is still sensitive to material hardening. This is due to the separate effect that aging concrete has on the contact time between the bottom particle of the chain and the interface, and on the repulsive force that determines the speed of the reflected waves. In fact, the TOF measured at a certain sensor particle consists of three components. The first is the travelling time of the incident wave between the sensor bead used for the measurement and the interface. This time depends on the impact energy of the striker and on the gravitational precompression. Thus, this travelling time is expected to be constant irrespective of the concrete's age. The second component of the TOF accounts for the contact time between the last bead and the testing material. This contact time is strongly affected by the stiffness of the material.

As the concrete gains strength, the contact time decreases. Based upon the values shown in Figures 6 and 7, it can be demonstrated that this second part of the TOF accounts for more than 50% of the TOF measured by the sensor bead when the concrete is fresh. Finally, the last part accounted in the TOF is the travelling time of the reflected pulse from the interface to the sensor bead. This travelling time is dependent on the particles pre-compression and the repulsory force generated at the interface. As the concrete gains strength, the reflected PSW and SSW are expected to increase their velocity and therefore the associated TOF is expected to diminish.

In order to quantify the effect of concrete age on the pulse amplitude, Figure 8 is presented. Figure 8a,b shows the ARP and the ARS as a function of the cement age as measured by the top and bottom sensor particle, respectively. While the amplitude ratio of the primary wave increases with concrete age (green cross marker), the ARS exhibits a relatively complex and inconclusive behavior (blue circles). By comparing the results shown in Figure 8 with the resistant pressure shown in Figure 6, it is worth noting that the amplitude of the primary reflected wave has a three-fold increase within the first 300 min and then it flattens when the initial set occurred. As such, the amplitude of the primary reflected wave might also be used to determine the initial set of concrete. More tests are necessary to validate such evidence.

## 4. Discussion and Conclusions

This article shows the working principles of a transducer able to generate and detect highly nonlinear solitary waves (HNSWs) and applied to monitor the hydration of fresh concrete. The transducer consists of a chain of spherical particles with instrumented beads, while an electromagnet is used for the generation and detection of the HNSWs. This transducer is used in a preliminary study aimed at developing a novel nondestructive testing method to estimate the initial and final set of fresh concrete and the temporal strength/stiffness development of the concrete. To prove the feasibility of the methodology, the transducer was used to monitor one concrete sample during hydration. It was found that a single pulse perpendicularly incident to the actuator/cement interface induces two reflected pulses, namely the primary solitary wave (PSW) and the secondary solitary wave (SSW). The amplitudes and the time-of-flight (TOF) of these two reflected waves are found to be strongly dependent on the stiffness of the sample. The trend of the HNSWs characteristics are compared with the penetration resistance obtained from the ASTM C 403 performed on mortar samples sieved from the concrete mixture. As the concrete became stiffer, the TOF of both the PSW and SSW decreased continuously. Two transition points were observed in the trend for the TOF with time. These points approximately corresponded to the times of initial and final set established using the ASTM C403 test.

Although the results are encouraging, more experiments are necessary to generalize the proposed methodology and to determine the relationship between HNSW parameters and the mechanical properties of material, which can then be related to penetration resistance from ASTM C403. First of all, the repeatability must be investigated by testing several samples of the same concrete batch and concrete samples with different water/cement ratios. Then, the effect of the spherical particles' size and material on the determination of the concrete properties should be studied to evaluate any effect on the prediction of the initial and final set. For instance, by enlarging the particles size the spatial wavelength increases, and the wave speed and amplitude decrease. By augmenting either the mass of the striker or the precompression force, both the amplitude and the wave speed increase. Future studies may also focus on finding the optimal design of the non-linear medium to maximize the sensitivity of the proposed technology to the changes of the concrete mechanical characteristics. This study would determine, for example, if these transducer parameters affect the set time determined using the HNSW device. Finally, a comparative study between the HNSW-based technology and current methodologies, such as those based on the use of bulk waves and the Schmidt hammer, should be carried out to quantify advantages and limitations of the proposed technique.

If the results found in this study are confirmed by the comprehensive studies summarized above, the novel nondestructive approach and the transducer described could provide some advantages over other conventional nondestructive testing methods based on linear ultrasonic bulk waves. In fact, the present approach uses only one transducer (instead of at least two) and does not require accessibility to the back-wall. With respect to the wave reflection method, where only the reflection coefficient is affected by the cement age, the present approach can virtually exploit three parameters: (1) the TOF of the primary reflected waves, (2) the TOF of the secondary reflected waves, and (3) the amplitude of the reflected waves. Moreover the HNSW-based method does not require the use of electronics for the generation of high-voltage input signals, contrary to piezoelectric transducers. It is acknowledged that the method presented in this paper implies that hydration is uniform in the whole material, by providing “effective” materials properties near the surface. If the hydration conditions are such that the mechanical properties of the material in the near field, *i.e.*, close to the actuator, are significantly different than in the far field, the HNSWs-based features may not be representative of the whole structure. Compared to the Schmidt hammer, which performs under a similar principle, the HNSW approach is nondestructive and robust in terms of pulse repeatability. Furthermore, the HNSW approach has more features to exploit and can be used to estimate “soft” materials such as fresh concrete. Overall, the preliminary results discussed show that the proposed technique has promising potential in characterizing the time-dependent strength-development of concrete. In the experiment conducted in this study a thin aluminum sheet was located between the chain of particles and the concrete, to prevent the free falling of the granules into the fresh concrete. Future studies should consider the effect of the sheet thickness on the repeatability and effectiveness of the proposed methodology. Alternatively the tube containing the particles can be tapered at the bottom in order to guarantee the contact of the last particle of the chain and the concrete without the free fall of the granules inside the fresh concrete. Future studies may consider particles of larger diameter or magnetostrictive sensors or piezo cylinders [45] for the measurement of the solitary waves.

## Figures and Tables

**Table 1.** Summary of the PCC mixture design used for the test.

**Materials**	**Batch Weight (kg/m^3^)**
Cement (Type I)	345
Fly Ash (Class C)	12
Water	145
Fine aggregate	744
Coarse aggregate (#57 Gravel)	1008
